# Exploring the Link: A Systematic Review and Meta‐Analysis on the Prevalence and Association Between Refractive Errors and Intermittent Exotropia

**DOI:** 10.1002/hsr2.70296

**Published:** 2024-12-19

**Authors:** Najah K. Mohammad, Ibrahim Ali Rajab, Mohammed Tareq Mutar, Mustafa Ismail

**Affiliations:** ^1^ Department of Surgery College of Medicine, University of Baghdad Baghdad Iraq

**Keywords:** intermittent exotropia, myopia, pediatric ophthalmology, refractive errors

## Abstract

**Background and Aims:**

Refractive errors and intermittent exotropia are prevalent conditions in pediatric populations, impacting visual development and quality of life. Despite the co‐occurrence of conditions such as myopia, hypermetropia, and astigmatism with strabismus, comprehensive analyses of their coexistence are limited. This study aims to investigate the prevalence and characteristics of refractive error among children with intermittent exotropia and find the correlation between the angle of deviation for far and near with factors like mean spherical equivalent and age.

**Methods:**

A systematic review and meta‐analysis were conducted in accordance with PRISMA guidelines. PubMed and Scopus databases were searched up to February 13, 2024. Inclusion criteria encompassed studies detailing clinical presentation, management strategies, and outcomes, while exclusion criteria eliminated review articles, conference abstracts, animal research, and studies with inadequate clinical information. Data extraction and quality assessment were independently performed by multiple reviewers, with discrepancies resolved through consensus. Forest plot was used to show the mean spherical equivalent and the angle of deviation, while publication bias was demonstrated using funnel plot with Egger test.

**Results:**

The search yielded 932 articles, with 23 meeting the inclusion criteria. Studies represented four geographical regions, with a combined sample size of 5407 participants aged 3–10 years. The mean refractive errors varied widely, with an overall pooled mean of −1.787 (95% CI: −5.392 to 1.818). Overminus lens therapy and surgical interventions were common management strategies, with surgery prevalent in cases with higher distant angle deviation. The myopia was more prevalent than hypermetropia (26.82%/16.10%). Meta‐regression showed that the mean spherical equivalent had a significant effect on the angle of deviation far and near.

**Conclusion:**

There was a significant correlation between myopia and intermittent exotropia in Middle Eastern and Asian pediatric populations. Myopia is notably more prevalent with significant effect of mean spherical equivalent on near‐angle deviation.

## 1 Introduction

The prevalence of refractive errors and intermittent exotropia (IXT), particularly, within pediatric populations, has increasingly become a focal point of ophthalmic research due to its significant impact on visual development and quality of life [1]. Common conditions such as myopia, hypermetropia, and astigmatism have been proved to be coexistent with a set of strabismic disorders. However, up to this date, there is no such number of literature reviews, which put to analysis these issues together in terms of their coexistence, if any. The most common was IXT—a condition in which one or both eyes turn outward, present in an intermittent manner [1, 2, 3].

Refractive errors are thus considered to have a complex relationship with IXT; this is an important area to be investigated further, as it has been evoked by central studies of Kushner and Rowe et al. [1, 4]. These works are salient in underscoring the necessity for focused research in the subtleties of the epidemiological and clinical dynamics in the two conditions. Kushner et al. have specifically addressed the impacts of overcorrecting minus lens therapy in myopic patients with IXT, pointing toward the need for a more profound understanding of treatment outcomes in this patient subset [1, 5].

With the paucity of good quality, comprehensive information regarding the prevalence of refractive error and its relationship with IXT in particular, from diverse geographic and ethnic variations, our study seeks to fill this gap. This study, therefore, attempts to fill the knowledge gap and shall analyze the prevalence and nature of refractive errors in children with IXT, particularly, delving into the investigation of the correlation between refractive error‐mean spherical equivalent and the angle of deviation at different distances.

## 2 Methods

### 2.1 Literature Search

This study presents an overview of the literature and meta‐analysis, in accordance with the PRISMA guidelines [6]. The databases PubMed and Scopus were thoroughly searched from their inception up to February 13, 2024. “Refractive errors,” OR “Myopia,” OR “Hyperopia,” OR “Astigmatism”,” AND “Intermittent exotropia,” OR “Divergent strabismus,” OR “Exodeviation,” OR “Squint” AND “pediatric,” OR “child*,” OR “infant*,” OR “newborn*,” OR “adolescent*,” OR “youth,” were the search terms combined by both Boolean operators “OR” and “AND.” Full texts of the studies identified were later imported into Rayyan for doing systematic reviews, where duplicate records were eliminated to ascertain accuracy and reliability of the data in analysis.

### 2.2 Study Selection

The criteria for including and excluding studies were established beforehand. Studies were eligible for inclusion if detailed information in regard to clinical presentation (the use of prism reflection or cover–uncover test for angle measurement, and the use of retinoscopy or auto refractometer for refractive error measurement), management strategies, and outcomes of the patients were well elaborated, and if they were published in English. On the other side, the exclusion criteria based on the PRISMA tool review of studies were those that reported (1) review articles, conference abstracts, animal research, cadaveric studies, and/or autopsy reports or (2) inadequate information in the studied articles for clinical characteristics, management methods, and/or outcomes of either one of the two or more. In case of overlapping patient cohorts, the study to be included in this analysis was only that which reported the duration of follow‐up.

Abstract screening and initial title screening of all the retrieved articles were independently done by two reviewers (I.A.R. and M.I.). They then independently screened titles and abstracts of articles meeting inclusion criteria, with discrepancies being resolved through consensus by the third reviewer (M.T.M.). Studies meeting the criteria for the review were included, and reference lists searched for further relevant studies.

### 2.3 Data Extraction

Data extraction was independently done by two reviewers (I.A.R. and M.I.), while completeness and accuracy of the extracted data were checked by a third reviewer (M.T.M.). No trial reviewed was reported to have any missing data by the authors. Extracted data included: Author and year of the study, country, study design, age, gender, sample size, refractive error range, spherical equivalent mean, sequent degree, type of exotropia, refractive error measurements techniques, key outcome measures, interventions, length of follow‐up, limitations, and level of evidence.

### 2.4 Data Synthesis, Quality Assessment, and Statistical Analysis

For this study, the primary focus was on evaluating the refractive error by evaluating the mean spherical equivalent and the angle of deviation both at near and distance in exotropia patients. Data synthesis involved this key outcome alongside other relevant clinical parameters. Study quality was assessed for the upgrade of the credibility of the results by two independent reviewers (I.A.R. and M.I.). The criteria of the adopted assessment were the level of evidence of each publication by the 2011 Oxford Center For Evidence‐Based Medicine recommendations [7, 8]. The Joanna Briggs Institute instruments were used to evaluate the risk of bias in the selected studies. Tools, such as the Joanna Briggs Institute checklists, have been prepared for use while analyzing various evidence, from case reports up to series. In terms of data presentation, continuous variables were summarized using medians and ranges, while categorical variables were expressed as frequencies and percentages.

These data were analyzed using JASP software (Version 0.19), with forest plot for mean spherical equivalent, and angle of deviation for distant and near, using the Maximum likelihood method. Meta‐regression was applied to investigate the effect of age, gender, mean spherical equivalent, refractive status proportion, and IXT subtypes on the angle of deviation. The sensitivity analysis and publication bias were evaluated with funnel plot and Egger test. A two‐tailed *p*‐value less than 0.05 is an indicator of statistical significance.

## 3 Result

**Figure 1:**
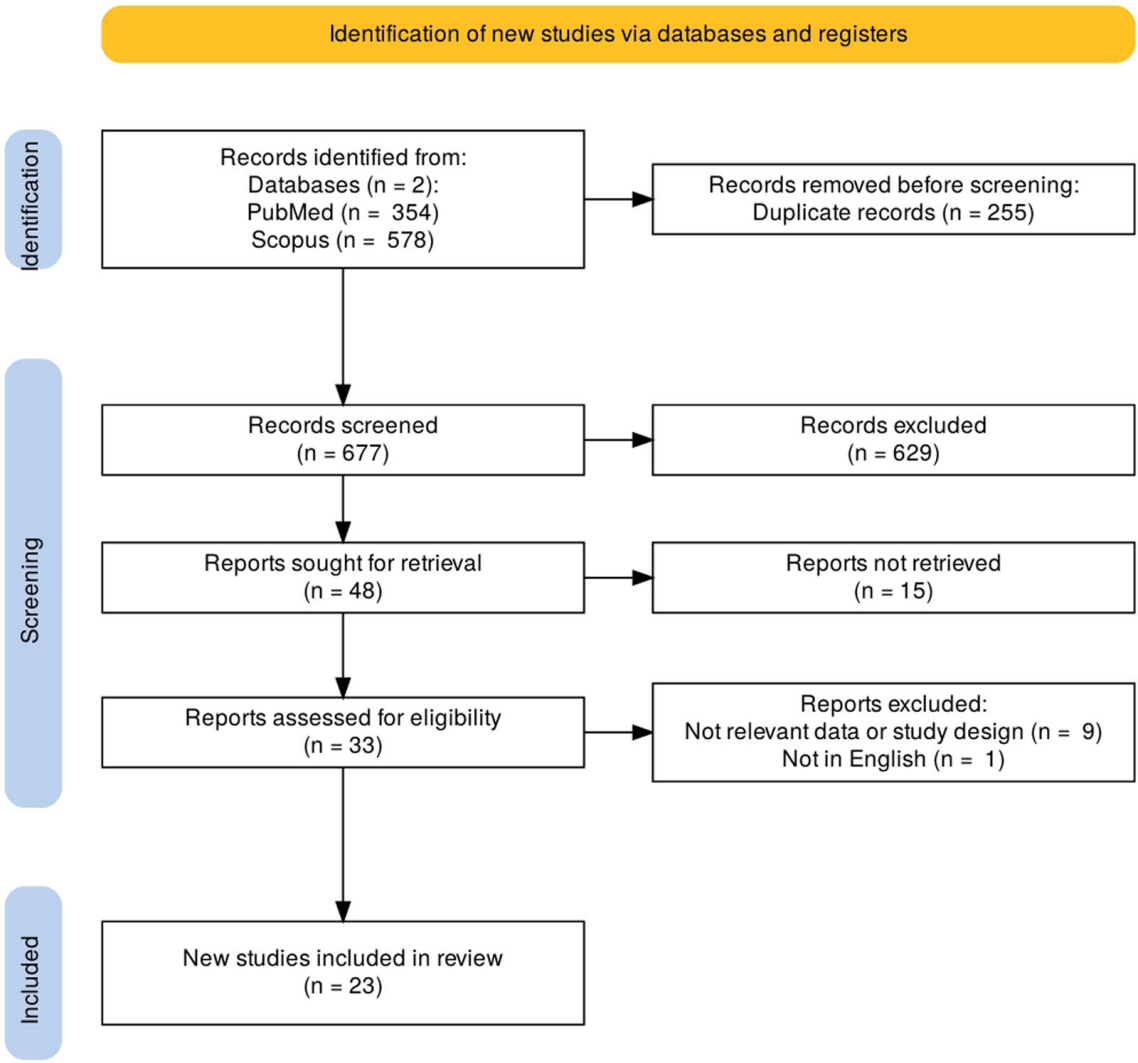
PRISMA flowchart.

The process of selecting studies for inclusion in this research was meticulously outlined in a PRISMA flowchart (Figure 1). The literature search process in this study revealed a total of 932 articles from the initial search process. The review of the abstracts resulted in the removal of duplicates and abstracts of articles with studies that are nonrelevant to this research. The review process, however, resulted in 33 articles that have studies possibly pertaining to the formulated research question. Twenty‐three articles were finally considered relevant and included in this systematic review and meta‐analysis following in‐depth assessment of the full texts of these articles (Supporting Information S1: File 1) [1, 2, 3, 4, 5, 9, 10, 11, 12, 13, 14, 15, 16, 17, 18, 19, 20, 21, 22, 23, 24, 25, 26].

In assessing the risk of bias, each included study was evaluated using The Joanna Briggs Institute instruments. The results indicated that most studies had a low risk of bias (Supporting Information S1: File 2).

These studies represented four geographical regions, with 18 from East Asia, 2 from Iran, 2 from the United States, and 1 from the United Kingdom. The sample sizes in the included studies ranged from 17 to 1228, with a combined total of 5407 participants. The age range of the included patient was (3–10) years. Seven of the included studies showed male predominance (50.4–60.7) and the remaining studies showed female predominance (50.6towards80), 15 of the included studies were retrospective studies.

The mean refractive errors varied widely across studies, ranging from –1.73 to 1.34, The overall pooled mean across studies was determined to be –1.787 (95% CI: –5.392 to 1.818).

Three studies evaluated overminus lens therapy while 13 studies focused on surgery for management with IXT and those patients had higher distant angle deviation than others.

**Figure 2:**
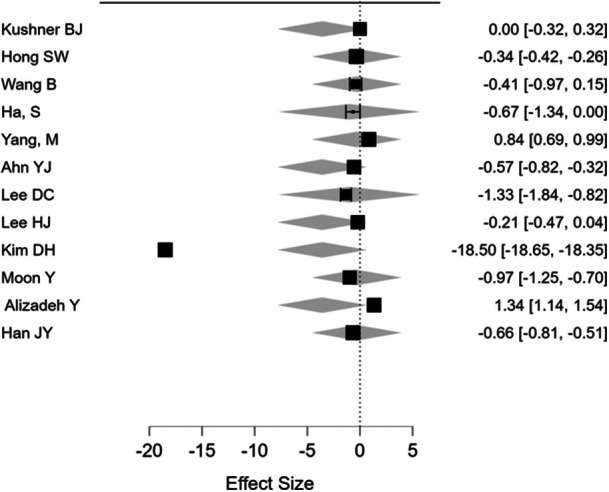
Forest plot of mean spherical equivalent.

**Figure 3:**
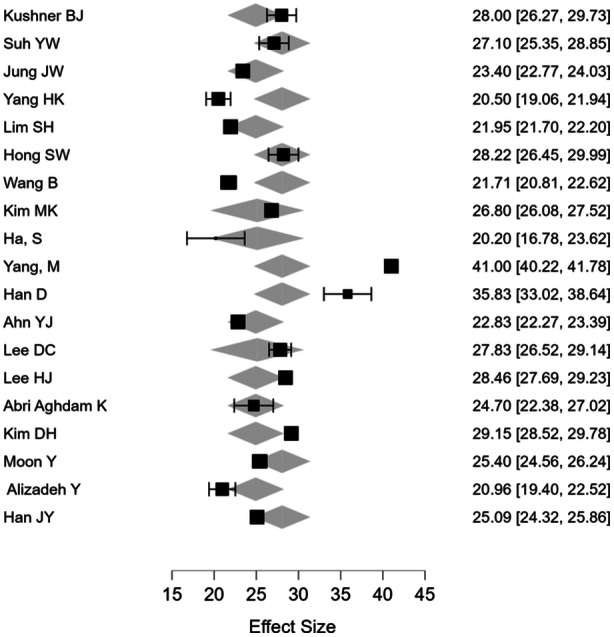
Forest plot of near‐distant angle deviation.

The pooled distant angle of deviation was (26.27) with a range of (21.71–‐41) and the mean near‐angle deviation was (30.52) with a range of (24–40) (Figures 2 and 3). The pooled proportion of myopia across studies was 26.82% (CI: 24.86%–28.78%) and for hypermetropia was 16.10% (CI: 14.46%–17.74%) (Tables 1, 2, 3).

**Table 1:** Mean refractive error.

Author	Year	Mean	Standard	Variance	Lower limit	Upper limit	*Z*‐value	*p*‐value
Kushner BJ	1999	0	0.163	0.026	−0.319	0.319	0	1
Hong SW	2012	−0.34	0.042	0.002	−0.423	−0.257	−8.069	0
Wang B	2014	−0.409	0.287	0.082	−0.972	0.154	−1.425	0.154
Ha S‐G	2016	−0.67	0.342	0.117	−1.34	0	−1.959	0.05
Yang M	2016	0.84	0.077	0.006	0.69	0.99	10.943	0
Lee D; Kim	2019	−1.73	0.41	0.168	−2.533	−0.927	−4.221	0
Ahn YJ	2019	−0.572	0.127	0.016	−0.821	−0.323	−4.51	0
Lee DC	2020	−1.33	0.259	0.067	−1.838	−0.822	−5.134	0
Lee HJ; Kim	2020	−0.215	0.13	0.017	−0.469	0.04	−1.649	0.099
Kim DH	2021	−18.5	0.079	0.006	−18.654	−18.346	−235.371	0
Moon Y	2023	−0.975	0.143	0.02	−1.254	−0.696	−6.839	0
Alizadeh Y	2023	1.34	0.104	0.011	1.136	1.544	12.894	0
Han JY	2023	−0.662	0.076	0.006	−0.811	−0.513	−8.705	0
Random	—	−1.787	1.839	3.384	−5.392	1.818	−0.971	0.331
Pred Int	—	−1.787			−16.929	13.355	—	—

**Table 2:** Distant deviation angle.

Author	Year	Standard	Mean	Variance	Lower limit	Upper limit	*Z*‐value	*p*‐value
Kushner BJ	1999	0.883	28	0.781	26.268	29.732	31.693	0
Suh YW	2006	0.892	27.1	0.795	25.352	28.848	30.393	0
Jung JW	2010	0.321	23.4	0.103	22.77	24.03	72.784	0
Yang HK	2011	0.737	20.5	0.543	19.055	21.945	27.814	0
Lim SH	2012	0.129	21.95	0.017	21.697	22.203	169.927	0
Hong SW	2012	0.904	28.22	0.817	26.448	29.992	31.213	0
Wang B	2014	0.461	21.714	0.212	20.811	22.617	47.109	0
Kim MK	2015	0.369	26.8	0.136	26.077	27.523	72.668	0
Ha S‐G	2016	1.744	20.2	3.041	16.782	23.618	11.584	0
Yang M	2016	0.4	41	0.16	40.217	41.783	102.625	0
Han D	2018	1.436	35.83	2.061	33.016	38.644	24.957	0
Ahn YJ	2019	0.287	22.829	0.083	22.266	23.392	79.435	0
Lee DC	2020	0.666	27.83	0.444	26.524	29.136	41.77	0
Lee HJ; Kim	2020	0.393	28.46	0.154	27.69	29.23	72.415	0
Abri	2021	1.183	24.7	1.399	22.382	27.018	20.884	0
Kim DH	2021	0.319	29.15	0.102	28.525	29.775	91.366	0
Moon Y	2023	0.429	25.4	0.184	24.56	26.24	59.267	0
Alizadeh Y	2023	0.796	20.96	0.634	19.399	22.521	26.317	0
Han JY	2023	0.395	25.089	0.156	24.316	25.862	63.587	0
Random	—	1.155	26.273	1.334	24.01	28.537	22.75	0
Pred Int	—		26.273		15.5	37.047	—	—

**Table 3:** Near deviation angle.

Author	Year	mean	Standard error	variance	Lower Limit	Upper Limit	*Z*‐value	*p*‐value
Suh YW	2006	30.6	0.947	0.896	28.745	32.455	32.326	0
Jung JW	2010	23.6	0.416	0.173	22.784	24.416	56.681	0
Yang HK	2011	42.5	1.795	3.222	38.982	46.018	23.678	0
Wang B	2014	33.51	0.661	0.437	32.214	34.806	50.697	0
Kim MK	2015	26.5	0.474	0.225	25.571	27.429	55.905	0
Ha S‐G	2016	21	1.945	3.784	17.188	24.812	10.796	0
Yang M	2016	40	0.457	0.208	39.105	40.895	87.607	0
Han D	2018	36.89	0.85	0.722	35.225	38.555	43.421	0
Ahn YJ	2019	24	0.384	0.148	23.247	24.753	62.485	0
Lee HJ; Kim	2020	33.49	0.384	0.148	32.737	34.243	87.165	0
Kim DH	2021	29.55	0.336	0.113	28.892	30.208	87.96	0
Moon Y	2023	30.1	0.543	0.295	29.036	31.164	55.447	0
Han JY	2023	25.34	0.384	0.148	24.587	26.093	65.963	0
Random	—	30.526	1.517	2.302	27.552	33.5	20.12	0
Pred Int	—	30.526	18.181	42.872	—	—	—	—

**Figure 4:**
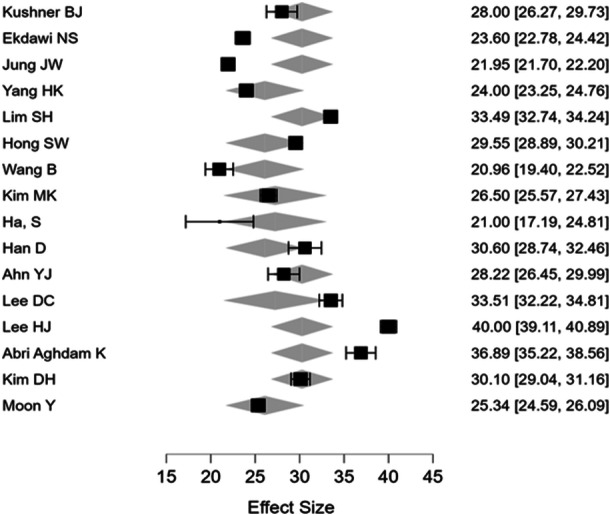
Forest plot. For near‐angle deviation.

**Figure 5:**
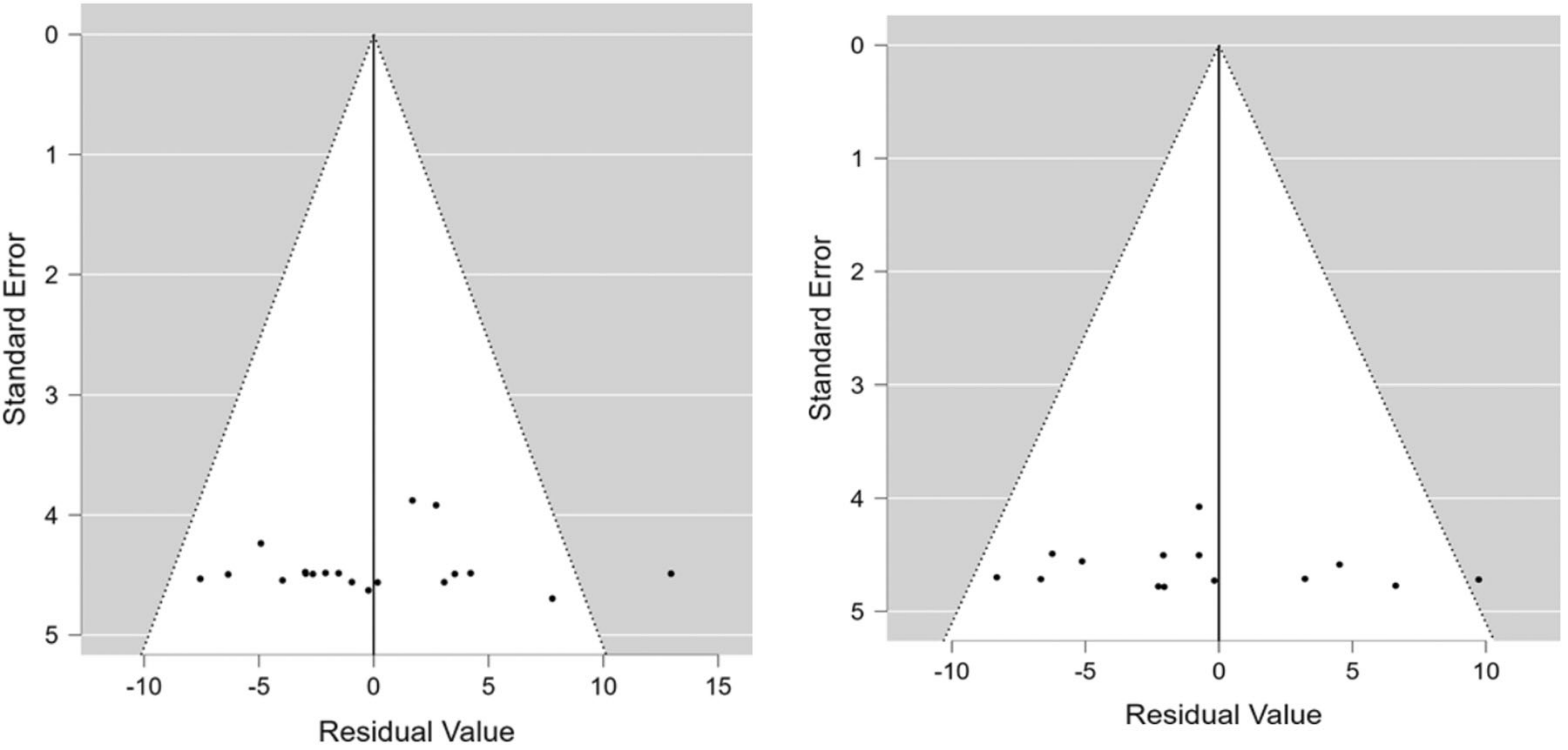
Funnel plot.

Meta‐regression for near and distant angle deviation showed that age and the combine age and mean spherical equivalent were the only significant predictors for near and distant angle deviation, whereas the mean spherical equivalent alone was significant for near deviation. The proportion of refractive status, gender, study size, and IXT subtypes were nonsignificant (Tables 4 and 5). For publication bias assessment, funnel plot was symmetrical and egger test demonstrated lack of bias in the reported studies (Figures 3, 4, 5).

**Table 4:** Meta‐regression for Distant angle deviation.

	Estimate	Standard error	*p*‐value	Lower CI	Upper CI
Intercept	33.198	5.433	< 0.001	22.549	43.847
MSE	5.176	2.904	0.075	−0.515	10.868
Male	−13.881	11.116	0.212	−35.667	7.905
Sample Size	0.008	0.004	0.051	−1.811 × 10 − 5	0.016
Age groups (> 8)	−14.378	6.662	0.031	−27.436	−1.321
Age groups (1–6)	−3.338	2.216	0.132	−7.680	1.005
MSE × Age groups (> 8)	−16.562	6.332	0.009	−28.973	−4.151
MSE × Age groups (1–6)	−5.235	2.980	0.079	−11.075	0.605
Meta‐regression for refractive status					
Intercept	25.088	25.052	0.317	−24.012	74.188
Myopia	5.574	55.037	0.919	−102.296	113.443
Hypermetropia	−4.334	73.225	0.953	−147.852	139.184

**Table 5:** Meta‐regression for near‐angle deviation.

	Estimate	Standard error	*p*‐value	Lower CI	Upper CI
Intercept	36.093	8.410	< .001	19.609	52.576
Male	−9.688	17.362	0.577	−43.717	24.342
MSE	7.557	2.710	0.005	2.246	12.868
Age groups (> 8)	−5.573	4.564	0.222	−14.519	3.373
Age groups (1–6)	−5.447	2.502	0.029	−10.351	−0.544
MSE × Age groups (1–6)	−7.751	2.717	0.004	−13.075	−2.427
Meta‐regression for refractive status					
Intercept	24.919	26.535	0.348		
Myopia	7.389	58.295	0.899		
Hypermetropia	−3.782	77.584	0.961		

We assessed heterogeneity for each pooled outcome using the *I*² statistic. The *I*² for pooled mean spherical equivalent was 45%, which refers to moderate heterogeneity; in the case of distant and near‐angle deviation, it was 32% and 38%, respectively, pointing to low to moderate heterogeneity. Sensitivity analysis excluding studies judged as having a higher risk of bias yielded consistent results, confirming the robustness of the results. The meta‐regression analysis therefore indicated that age and mean spherical equivalent jointly and significantly predicted the overall near‐angle of deviation, *β* = 0.27, 95% CI: 0.12–0.42, *p* = 0.004; similarly, for the overall distant angle of deviation, the interaction effect of age and mean spherical equivalent was statistically significant, *β* = 0.18, 95% CI: 0.05–0.31, *p* = 0.01. The other factors such as gender, study size, and IXT subtypes were not significantly influencing the angle of deviation: *p* > 0.05 (Tables 4 and 5).

The Basic type of IXT was the most common subtype reported in all the studies with a reported prevalence ranging from 44.6% to 100%, with larger studies, reporting 88.1% [16]. Convergence Insufficiency was the second with a prevalence ranges from 2% to 26.4%, with the highest rate of 26.4% found in Suh et al. study [5]. Pseudo‐divergence excess prevalence generally falls between 5% and 10%, with some studies noting rates of 10.1% and 7.4% [3, 16].

## 4 Discussion

The outward deviation of one eye can either be latent or at times manifest for a transient period, sometimes referred to as IXT [27]. This condition occurs in up to 1%–5% prevalence rate and is one of the most common forms of strabismus in children [28].

The interpretation of results from the various clinical studies on the characteristics of IXT has been a huge task due to differences in study settings, protocols, and great clinical variability of the condition [29]. We report this review to determine the mean refractive error and the angles of deviation at near and distant viewing distances in patients with IXT.

Current management options for IXT are conservative treatment with watchful waiting, overminus spectacles, part‐time occlusion therapy, and surgical intervention. Overminus lens therapy is nonsurgical management used in the management of IXT in childhood [30]. This kind of therapy includes prescribing spectacles with stronger lens power for minifying compared to that which is determined by the cycloplegic refractive error. The idea is to stimulate accommodation, which in turn controls outward drifting of the eyes and helps in good alignment. In this study, three articles evaluated over‐minus therapy in IXT [1, 21, 23].

In this review surgery, was selected for management in 13 studies [9, 10, 11, 12, 13, 15, 17, 18, 19, 20, 22, 23, 25]. The mean age of surgery in this study was 6.096 (4.091–7.291). Studies that focus on the effect of age on surgery and outcomes present conflicting findings. While some research indicates a greater surgical response and higher incidence of overcorrection in younger age groups [31, 32, 33], other studies suggest no correlation between patient's age at surgery and outcomes [34, 35, 36, 37]. Studies have revealed that patients with persistent overcorrection are more likely to have a higher mean age at the time of the surgery than those who do not have persistent overcorrection [38].

The distant angle deviation ranges from 21.71 to 41 in those undergoing surgery while the distant angle deviation ranges from 20.96 to 24.70 in those treated with over‐minus lens therapy. This may reflect that patients with larger angle deviation are more likely to get benefits of surgery compared to those with milder degrees who may benefit from over‐minus lens therapy [10, 11, 28, 30, 31, 32, 33, 34, 35, 36, 37, 38, 39, 40].

The mean refractive error in this review is −1.788 (−4.768, 1.192) suggesting myopic predominance while the mean RE in surgical patients is −2.390 (−6.77, 1.990). Children with myopia exhibit a 5.23‐fold increase in the risk of developing exotropia compared to those without significant ametropia. This may elucidate the high prevalence of exotropia in Asia, where myopia rates are notably higher [41, 42]. Exotropia is more common in Asia, the Middle East, and Africa which supports the findings in this study as only two studies were done outside Asia (USA) [3].

As the eye shifts focus from far to near, accommodation, convergence, and pupillary constriction happen together. Depending on the fusional convergence and divergence strength, the angle of eye deviation changes between distant and close focus. The prism cover test is used to classify IXT into basic, simulated distance, or true distance types based on these deviation patterns [27]. Among the reviewed studies, the basic subtype was the most prevalent, followed by convergence insufficiency.

Current management options for IXT include conservative approaches like over‐minus spectacles, part‐time occlusion therapy, and surgical intervention [30]. Over‐minus therapy was examined in three studies [1, 21, 23], while surgery was the focus in 13 studies [9, 10, 11, 12, 13, 15, 17, 18, 19, 20, 22, 23, 25], with an average age of 6.1 years (range: 4.1–7.3 years). The distant angle deviation ranges from 21.71 to 41 in those undergoing surgery while the distant angle deviation ranges from 20.96 to 24.70 in those treated with over‐minus lens therapy. This may reflect that patients with larger angle deviation are more likely to get benefits of surgery compared to those with milder degrees who may benefit from over‐minus lens therapy [10, 11, 28, 30, 31, 32, 33, 34, 35, 36, 37, 38, 39, 40].

## 5 Conclusion

This review reveals IXT in Middle Eastern and Asian pediatric populations, with myopia being prevalent than hypermetropia in those patients The mean spherical equivalent and age were significant factor for angle deviation.

## Author Contributions

**Najah K. Mohammad:** methodology, data curation, supervision, writing–original draft, writing–review and editing. **Ibrahim Ali Rajab:** writing–review and editing, writing–original draft, conceptualization. **Mohammed Tareq Mutar:** conceptualization, writing–original draft, writing–review and editing, formal analysis, validation. **Mustafa Ismail:** conceptualization, writing–original draft, writing–review and editing, data curation, software, validation, investigation, visualization, project administration.

## Conflicts of Interest

The authors declare no conflicts of interest.

## Transparency Statement

The lead author Najah K. Mohammad affirms that this manuscript is an honest, accurate, and transparent account of the study being reported; that no important aspects of the study have been omitted; and that any discrepancies from the study as planned (and, if relevant, registered) have been explained.

## Supporting information

Supporting information.

Supporting information.

Supporting information.

## Data Availability

The summary of the included studies is available in the Supporting Information and further details are available upon request. The data sets generated and/or analyzed during the current study are available from the corresponding author on reasonable request. As this study is a systematic review and meta‐analysis, the data is already published in the literature.

## References

[1] B. J. Kushner , “Does Overcorrecting Minus Lens Therapy for Intermittent Exotropia Cause Myopia?,” Archives of Ophthalmology 117, no. 5 (May 1999): 638–642. 10326961 10.1001/archopht.117.5.638

[2] N. S. Ekdawi , K. J. Nusz , N. N. Diehl , and B. G. Mohney , “The Development of Myopia Among Children With Intermittent Exotropia,” American Journal of Ophthalmology 149, no. 3 (May 2010): 503–507. 20172074 10.1016/j.ajo.2009.10.009 PMC3926435

[3] J. W. Jung and S. Y. Lee , “A Comparison of the Clinical Characteristics of Intermittent Exotropia in Children and Adults,” Korean Journal of Ophthalmology 24, no. 2 (April 2010): 96–100. 20379459 10.3341/kjo.2010.24.2.96 PMC2851009

[4] F. J. Rowe , C. P. Noonan , G. Freeman , and J. DeBell , “Intervention for Intermittent Distance Exotropia With Overcorrecting Minus Lenses,” Eye 23, no. 2 (February 2009): 320–325. 18064054 10.1038/sj.eye.6703057

[5] Y. W. Suh , S. H. Kim , J. Y. Lee , and Y. A. Cho , “Conversion of Intermittent Exotropia Types Subsequent to Part‐Time Occlusion Therapy and Its Sustainability,” Graefe's Archive for Clinical and Experimental Ophthalmology 244, no. 6 (June 2006): 705–708. 10.1007/s00417-005-0195-0 16463040

[6] M. J. Page , J. E. McKenzie , P. M. Bossuyt , et al., “The PRISMA 2020 Statement: An Updated Guideline for Reporting Systematic Reviews,” International Journal of Surgery 88 (2021): 105906. 33789826 10.1016/j.ijsu.2021.105906

[7] J. Howick , I. Chalmers , P. Glasziou , et al., “Oxford Centre for Evidence‐Based Medicine Levels of Evidence Centre for Evidence‐Based Medicine, 2011, https://www.cebmnet/wp-content/uploads/2014/06/CEBM-Levels-of-Evidence-21-pdf.

[8] M. Assel , D. Sjoberg , A. Elders , et al., “Guidelines for Reporting of Statistics for Clinical Research in Urology,” BJU International 123, no. 3 (March 2019): 401–410, 10.1111/bju.14640. 30537407 PMC6397060

[9] H. K. Yang and J. M. Hwang , “Decreased Accommodative Response in the Nondominant Eye of Patients With Intermittent Exotropia,” American Journal of Ophthalmology 151, no. 1 (January 2011): 71–76.e1. 20970775 10.1016/j.ajo.2010.06.047

[10] S. H. Lim , B. S. Hwang , and M. M. Kim , “Prognostic Factors for Recurrence After Bilateral Rectus Recession Procedure in Patients With Intermittent Exotropia,” Eye 26, no. 6 (June 2012): 846–852. 22441025 10.1038/eye.2012.55 PMC3376299

[11] J. H. Jang , J. M. Park , and S. J. Lee , “Factors Predisposing to Consecutive Esotropia After Surgery to Correct Intermittent Exotropia,” Graefe's Archive for Clinical and Experimental Ophthalmology 250 (2012): 1485–1490. 10.1007/s00417-012-1991-y 22450527

[12] S. W. Hong and N. Y. Kang , “Astigmatic Changes After Horizontal Rectus Muscle Surgery in Intermittent Exotropia,” Korean Journal of Ophthalmology 26, no. 6 (December 2012): 438–445. 23204799 10.3341/kjo.2012.26.6.438 PMC3506818

[13] B. Wang , L. Wang , Q. Wang , and M. Ren , “Comparison of Different Surgery Procedures for Convergence Insufficiency‐Type Intermittent Exotropia in Children,” British Journal of Ophthalmology 98, no. 10 (October 2014): 1409–1413. 24842862 10.1136/bjophthalmol-2013-304442

[14] M. K. Kim , U. S. Kim , M. J. Cho , and S. H. Baek , “Hyperopic Refractive Errors as a Prognostic Factor in Intermittent Exotropia Surgery,” Eye 29 (2015): 1555–1560. 26293140 10.1038/eye.2015.152 PMC5129793

[15] S. G. Ha , S. M. Jang , Y. A. Cho , S. H. Kim , J. S. Song , and Y. W. Suh , “Clinical Exhibition of Increased Accommodative Loads for Binocular Fusion in Patients With Basic Intermittent Exotropia,” BMC Ophthalmology 16 (June 2016): 77. 27266700 10.1186/s12886-016-0260-y PMC4896026

[16] M. Yang , J. Chen , T. Shen , et al., “Clinical Characteristics and Surgical Outcomes in Patients With Intermittent Exotropia: A Large Sample Study in South China,” Medicine 95, no. 5 (February 2016): e2590. 26844467 10.1097/MD.0000000000002590 PMC4748884

[17] D. Han , D. Jiang , J. Zhang , T. Pei , and Q. Zhao , “Clinical Study of the Effect of Refractive Status on Stereopsis in Children With Intermittent Exotropia,” BMC Ophthalmology 18 (2018): 143. 29921242 10.1186/s12886-018-0822-2 PMC6011197

[18] D. Lee , M. Kim , W. J. Kim , and M. M. Kim , “Changes in Refractive Error and Axial Length After Horizontal Muscle Surgery for Strabismus,” Journal of American Association for Pediatric Ophthalmology and Strabismus 23, no. 1 (2019 Feb): 20.e1–20.e5. 10.1016/j.jaapos.2018.08.010 30582982

[19] Y. J. Ahn , Y. Y. Park , Y. W. Chung , S. H. Park , and S. Y. Shin , “Surgical and Sensory Outcomes in Patients With Intermittent Exotropia According to Preoperative Refractive Error,” Eye 33, no. 8 (August 2019): 1314–1320. 30932034 10.1038/s41433-019-0419-x PMC7005694

[20] D. C. Lee and S. Y. Lee , “Analysis of Astigmatism Outcomes After Horizontal Rectus Muscle Surgery in Patients With Intermittent Exotropia,” PLoS One 15, no. 10 (October 2020): e0240026. 33031390 10.1371/journal.pone.0240026 PMC7544045

[21] H. J. Lee and S. J. Kim , “Long‐Term Outcomes Following Resection‐Recession Versus Plication‐Recession in Children With Intermittent Exotropia,” British Journal of Ophthalmology 104, no. 3 (March 2020): 350–356. 31118183 10.1136/bjophthalmol-2018-313711

[22] K. Abri Aghdam , A. Zand , M. Soltan Sanjari , S. Khorramdel , and R. Asadi , “Overminus Lens Therapy in the Management of Children With Intermittent Exotropia,” Journal of Current Ophthalmology 33, no. 1 (March 2021): 36–40. 34084955 10.4103/JOCO.JOCO_17_20 PMC8102942

[23] D. H. Kim , H. K. Yang , and J. M. Hwang , “Long‐Term Surgical Outcomes of Unilateral Recession‐Resection Versus Bilateral Lateral Rectus Recession in Basic‐Type Intermittent Exotropia in Children,” Scientific Reports 11, no. 1 (September 2021): 19383. 34588536 10.1038/s41598-021-98801-3 PMC8481325

[24] Y. Moon and S. J. Kim , “Refractive Changes After Strabismus Surgery in Patients With Intermittent Exotropia,” PLoS One 18, no. 1 (January 2023): e0280274. 36634079 10.1371/journal.pone.0280274 PMC9836274

[25] Y. Alizadeh , A. Medghalchi , S. Soltanipour , et al., “Role of Overcorrecting Minus Lens Therapy in Intermittent Exotropia for Prevention of Constant Exotropia in Children Under the Age of 7 Years,” International Journal of Preventive Medicine 14 (June 2023): 80. 37854980 10.4103/ijpvm.ijpvm_130_22 PMC10580209

[26] J. Y. Han , J. Han , and S. H. Han , “Correlation Between Bilateral Lateral Rectus Muscle Recession and Myopic Progression in Children With Intermittent Exotropia,” Scientific Reports 13, no. 1 (May 2023): 7200. 37137972 10.1038/s41598-023-34441-z PMC10156685

[27] D. H. Kim , J. H. Jung , M. Y. Choi , et al., “A Cross‐Sectional Study of Ophthalmologic Examination Findings in 5385 Koreans Presenting With Intermittent Exotropia,” Scientific Reports 13 (2023): 1329. 36693891 10.1038/s41598-023-28015-2 PMC9873724

[28] B. W. Zhao , J. H. Wang , H. X. Bai , et al., “Quality of Life in Adult Intermittent Exotropia and the Risk Factors,” International Journal of Ophthalmology 14, no. 3 (March 2021): 442–447, 10.18240/ijo.2021.03.18. 33747823 PMC7930552

[29] A. K. C. Chiu , N. Din , and N. Ali , “Standardising Reported Outcomes of Surgery for Intermittent Exotropia: A Systematic Literature Review,” Strabismus 22, no. 1 (March 2014): 32–36. 24564726 10.3109/09273972.2013.877940

[30] A. M. Chen , S. A. Erzurum , D. L. Chandler , et al., “Overminus Lens Therapy for Children 3 to 10 Years of Age With Intermittent Exotropia: A Randomized Clinical Trial,” JAMA Ophthalmology 139, no. 4 (April 2021): 464–476. 33662112 10.1001/jamaophthalmol.2021.0082 PMC7934080

[31] A. B. Scott , A. J. Mash , and A. Jampolsky , “Quantitative Guidelines for Exotropia Surgery,” Investigative Ophthalmology 14 (1975): 428–436. 1132940

[32] P. M. Edelman , M. H. Brown , A. L. Murphree , and K. W. Wright , “Consecutive Esodeviation, Then What,” American Orthoptic Journal 38 (1988): 111–116.

[33] Y. J. Gordon and E. Bachar , “Multiple Regression Analysis Predictor Models in Exotropia Surgery,” American Journal of Ophthalmology 90 (1980): 687–691. 7446649 10.1016/s0002-9394(14)75138-4

[34] B. J. Kushner , “Factors Influencing Response to Strabismus Surgery,” Archives of Ophthalmology 111 (1993): 75–78. 8424728 10.1001/archopht.1993.01090010079030

[35] O. E. Abbasoglu , E. C. Sener , and A. S. Sanac , “Factors Influencing the Successful Outcome and Response in Strabismus Surgery,” Eye 10 (1996): 315–320. 8796155 10.1038/eye.1996.66

[36] J. M. Richard and M. M. Parks , “Intermittent Exotropia,” Ophthalmology 90 (1983): 1172–1177. 6657192

[37] E. A. Dunlap , “Overcorrections in Exotropia Surgery,” in Symposium on Horizontal Ocular Deviations, ed. D. R. Manley (St Louis: Mosby, 1971), 183.

[38] R. V. Keech and S. A. Stewart , “The Surgical Overcorrection of Intermittent Exotropia,” Journal of Pediatric Ophthalmology & Strabismus 27 (1990): 218–220. 2391624 10.3928/0191-3913-19900701-12

[39] S. Tibrewal , N. Singh , M. I. Bhuiyan , and S. Ganesh , “Factors Affecting Residual Exotropia After Two Muscle Surgery for Intermittent Exotropia,” International Journal of Ophthalmology 10 (2017): 1120–1125. 28730116 10.18240/ijo.2017.07.16 PMC5514275

[40] D. Zou , C. Casafina , A. Whiteman , and S. Jain , “Predictors of Surgical Success in Patients With Intermittent Exotropia,” Journal of American Association for Pediatric Ophthalmology and Strabismus 21 (2017): 15–18. 28089744 10.1016/j.jaapos.2016.11.018

[41] D. Robaei , A. Kifley , and P. Mitchell , “Factors Associated With a Previous Diagnosis of Strabismus in a Population‐Based Sample of 12‐Year‐Old Australian Children,” American Journal of Ophthalmology 142 (2006): 1085–1087.e1. 17157605 10.1016/j.ajo.2006.06.053

[42] G. Wen , K. Tarczy‐Hornoch , R. McKean‐Cowdin , et al., “Prevalence of Myopia, Hyperopia, and Astigmatism in Non‐Hispanic White and Asian Children,” Ophthalmology 120 (2013): 2109–2116. 23953098 10.1016/j.ophtha.2013.06.039 PMC3902090

