# Supplementary material for: Exploring the Link: A Systematic Review and Meta‐Analysis on the Prevalence and Association Between Refractive Errors and Intermittent Exotropia
Source: Health Sci Rep. 2024 Dec 19;7(12):e70296. doi: 10.1002/hsr2.70296 (PMC11659191; doi:10.1002/hsr2.70296)
Supplement: Supplementary file 1 — Supporting information. [file HSR2-7-e70296-s001.docx]

|  |  |  |  |  |  |  |  |  | Refractive error defintion (range) | | | | | | | Spherical Equivalent mean, SD | Squint degree (PD) mean+ SD | | Type of exotropia N,% | | | | | |  |  |  |  |  |  |  | Repeated-measures ANOVA, independent t test | | |
| --- | --- | --- | --- | --- | --- | --- | --- | --- | --- | --- | --- | --- | --- | --- | --- | --- | --- | --- | --- | --- | --- | --- | --- | --- | --- | --- | --- | --- | --- | --- | --- | --- | --- | --- |
| Study ID | **Author** | **Year of Publication** | **Country** | **Study Design** | **Age (Mean+SD) OR (Median+IQR)** | **Gender Male %** | **Sample Size (n)** | **Diagnostic Criteria for Intermittent Exotropia** | **Myopia** | **Hypermetropia** | | **Astigmatism** | | **Emmetropia** | |  | **Distant deviation (PD)** | **Near deviation** | **Basic** | **Divergence excess** | **Convergence insufficiency** | | **pseudo-divergence excess** | | **Type of Refractive Error Evaluated (ex. Myopia N, %)** | **Refractive Error Measurement Techniques** | **Key Outcome Measures** | **Intervention(s) (if applicable)** | **Length of Follow-up** | **Statistical Analysis Used** | **Main Findings** | **Limitations** | | **Level of Evidence** |
| 1 | Kushner BJ (1) | 1999 | USA | Retrospective chart review | 4.1±1.5 | 40% | 74 |  |  |  | |  | |  | |  |  |  |  |  |  | |  | | myopia |  |  |  | 6 months and 5 years | t-test | Overcorrecting minus lens therapy | | | |
| 2 | Suh YW (5) | 2006 | Republic of Korea | Prospective | 6.41±3.3 | 47% | 70 |  |  |  | |  | |  | |  | 27.1±7.46 | 30.6±7.92 | 44,63% |  | 26,37% | |  | |  |  |  | patching | 3 months | t-test |  |  |  | |
| 3 | Rowe FJ (4) | 2009 | UK | Prospective cohort study | 5 ±2.2 | 38% | 21 |  |  |  | |  | |  | |  |  |  |  |  |  | |  | | myopia, –1.54± –1.80 | |  |  | 5 years | wilcoxon and t test | Overcorrecting minus lens therapy | Small sample, unable to obtain annual refraction information, problematic cooperation and compliance. | | |
| 4 | Ekdawi NS (2) | 2010 | USA | Retrospective population based observational study | 5.6 years (range, 0.9 to 14.9 years). | 33% | 135 | Intermittent distance exodeviation of at least 10 PD without associated neurologic, paralytic, or anatomic disorder. | more than or equal to –0.50 diopters | | | | |  | |  | 20 PD (range, 10 to 40 PD) | 14 PD (range, 0 to 45 PD) | |  |  | |  | |  |  |  |  | 20 years | Wilcoxon rank and Fisher exact tests | | Imprecise inclusion criteria and unequal follow-up, not all refractions were performed with a cycloplegic agent, could not determine the precise age at myopia onset, unable to identify a representative control group from the same population with which to compare our refractive error findings, might be selection bias. | | |
| 5 | Jung JW (9) | 2010 | Korea | Retrospective study was conducted using the medical records | 5.1±3.8 | 52.20% | 360 |  | (>2 D) | (>1 diopters [D]) | (>1.5 D) | | |  | |  | 23.4±6.1 | 23.6±7.9 | 310 (86.2%) | 2 (0.5%) | 12 (3.2%) | | 36 (10.1%) | | Astigmatism 268 (41.0%) , Myopia 184 (28.2%) , Hyperopia 163 (25.0%) | | | | | Chisquare test and T-test | | Lower compliance rate in the pediatric patient group | | |
| 6 | Yang HK (10) | 2011 | Korea | Observational cohort study. | 7.1± 5.4 | 20% | 133 | 20.55 |  |  |  | | |  | |  | 20.5 ± 8.5 | 42.5±20.7 |  |  |  | |  | |  |  |  |  |  | Pearson chi-square test, Fisher exact test, and one-way analysis of variance | | Strict inclusion criteria, lack information on the accommodative response to distant targets. | | |
| 7 | Lim SH (11) | 2012 | Korea | Retrospective review of the medical records | 3.01±2.09 | 47.00% | 511 |  |  |  |  | | |  | |  |  |  |  |  |  | |  | |  |  |  |  | 12 months | Multivariate logistic regression test, Kaplan–Meier survival test, and the independent t-test and chi square analysis were used, | | Major limitation of this study is its nonrandomized, retrospective design. A second limitation is the lack of sensory test analysis. | | |
| 8 | Jang JH (12) | 2012 | Korea | Retrospective survey | 7.1±5.9 | 45.13% | 266 |  |  |  |  | | |  | |  |  |  | 177 (66.5%) |  | 26 (0.1%) | |  | |  |  |  |  | 1 year | Pearson’s chi-squared test | |  |  | |
| 9 | Hong SW (13) | 2012 | Korea | Retrospective review | 7.59 ± 2.92 years | 36.20% | 69 |  |  |  |  | | |  | |  |  |  |  |  |  | |  | |  |  |  |  | 6 months | Student t-test, Repeated measures ANOVA (RMANOVA) and paired t test | | | | |
| 10 | Wang, B (14) | 2014 | Republic of China | Prospective surgical study | 6.17± 2.09 | 53% | 45 | Patients with CI-type IXT; age 3-15 years; best-corrected visual acuity in the worse eye 20/40 or better and exodeviation at distance between 15 and 25 PD by PACT with appropriate optical correction. | | | | | | | | | | | | | | | | | | | | | 6 months | Univariate ANOVA repeated measures ANOVA chi square test | | Small sample size, follow-up period relatively short. | | |
| 11 | Kim MK (15) | 2015 | Korea | Retrospective review of the medical records | 8.4 ± 4.9 | 54% | 333 |  |  |  |  | |  | |  | | 26.8 ± 6.73 | 26.5 ± 8.65 | 282(84%) | 8(0.02%) | 17(0.05%) | 26(0.07) | |  | |  |  |  | 6 months | (ANOVA), Chi-square test, and repeated measures ANOVA were | hyperopic refractive error was a good prognostic factor in IXT surgery in this patient population | There is variability within patients population. The sample size of the hyperopia group was much smaller than that in the other groups and thus the /age and follow-up time ranges are wide, and the age effect could not be properly accounted for. stereopsis was evaluated using only near stereoauity measures (Titmus test) in this study. | | |
| 12 | Ha, S.-G (16) | 2016 | South Korea | Case control | 10.0 ± 4.09 | 46.90% | 17 | Normal accommodative convergence/ accommodation ratio ; difference between long and short deviation <10 PD; lack of tenacious proximal fusion, AND lack of strong proximal convergence after monocular occlusion for 1 h. | | | | | | | | | 20.2 ± 7.19 | 21.0 ± 8.02 | 17(100%) |  |  |  | |  | | The WAM-5500 autorefractor | |  |  | Mann–Whitney U test, Chi-square test ,The Wilcoxonsinged rank test and Kruskal-Wallis test. | | The sample size was relatively small, the included patients were only diagnosed with the basic type of IXT. cycloplegic refraction was not performed for subjects who did not require glasses. | | |
| 13 | Yang, M (17) | 2016 | China | Retrospective review | 6.77± 6.43 | 50.40% | 1228 |  |  |  |  | |  | |  | | 41±14 | 40±16 | 1082 (88.1%) |  | 55 (4.5%) | 91(7.4%) | |  | |  |  |  | 7.8±3.7 months | t test or Mann–Whitney U test. Chi-square or Fisher exact tests. Multivariate logistic regression. | | | | |
| 14 | Han D (18) | 2018 | China | Retrospective review | 7.73 ± 2.83 | 53.30% | 90 |  | Spherical equivalent < or = − 1.00 diopters) | (spherical equivalent > or = − 1.00 diopters) | | | spherical equivalent diopter: within ±1.00DS | | | | 35.83 ± 13.62 | 36.89 ± 8.06 |  |  |  |  | | Hyperopia 15 (16.7%) Emmetropia 31 (34.4%) Myopia 26 (28.9%) Anisometropia 18 (20%) | | | | | | Chi square test, independent sample t-test | | First,the fact that 4 separate surgeons and 3 study examiners were used will introduce some variability in the data. Second, the retrospective nature. Third, the follow-up times were quite variable among individual cases and the average follow-up time was somewhat brief. Fourth, in many cases the onset of exotropia in this study was based on patient history, which can be subject to recall bias. geographical bias in our series. | | |
| 15 | Lee D (19) | 2019 | South Korea | Retrospective cohort study | 11.41±6.77 | 36% | 47 |  |  |  |  | |  | | −1.73 ± 2.81 | |  |  |  |  |  |  | |  | | autorefractor (Huvitz HRK-7000A, Huvitz Co Ltd, Gunpo, Gyeonggi-do, South Korea) | | | 12 months |  |  | IOP and corneal thickness were measured by noncontact tonometry. the age at surgery of patients in the RR group was significantly older than that of the LR group patients. selection bias | | |
| 16 | Ahn YJ (20) | 2019 | Republic of Korea | Observational case series | 4.85± 3.9 | 46.90% | 326 |  | SE ≤−1D | SE ≥+2D |  | | SE between −1D and +2D | | Hyperopic group: 2.93 ± 1.08  Emmetropic group: 0.34 ± 0.59  Myopic group: −2.82 ± 1.90 | | Hyperopic group: 24.26 ± 8.07 Emmetropic group: 22.68 ± 4.59 Myopic group: 22.64 ± 4.95 | Hyperopic group: 25.56 ± 8.95 Emmetropic group: 23.09 ± 6.72 Myopic group: 24.84 ± 6.46 | 297(91.1%) | 4(0.01%) | 7(0.02%) | 18(0.05%) | | hyperopic group 34 (10.4%) myopic group 122 (37.4%) emmetropic group 170 (52.1%) | | | | | 38.5± 25.3 months | (ANOVA), Chi-square test, and repeated measures ANOVA ,multivariate logistic regression. | | Intergroup variability exists follow-up period was relatively short. direct comparisons with other studies are limited. | | |
| 17 | Lee DC (21) | 2020 | Korea | Retrospective cross-sectional study | 9.55 ± 5.03 years | 49.40% | 83 |  |  |  |  | |  | | -1.33 ± 2.36 | |  |  |  |  |  |  | | Astigmatism | | Galilei G4 Dual Scheimpflug Analyzer (Ziemer, Port, Switzerland) | | | 3 months | Paired t tests were a univariable linear regression. | | Retrospective nature and a short followup, small sample size | | |
| 18 | Lee HJ (22) | 2020 | South Korea | Retrospective chart review | 3.1±1.9 years | 45.60% | 186 |  |  |  |  | |  | | 33.2±4.95 | | 28.25±5.3 |  |  |  |  |  | |  | |  |  |  | 35.05±10.6 months | Independent Student’s t-test, χ2 test and Fisher’s exact test. A logistic regression analysis. Kaplan Meier survival analysis and the log-rank test. | | Retrospective study. variations in the final follow-up period among patients. Patients with successful outcomes tend to stop visiting the hospital, while patients with Unfavourable results were followed up for a longer duration, resulting in higher recurrence rates. We also measured motor status by only alternate prism cover test. In addition, the age range of this study was quite wide. | | |
| 19 | Abri Aghdam (23) | 2021 | Iran | Retrospective study | 3 ± 2.6 years | 60.70% | 163 |  |  |  |  | |  | | 0.24 + | |  |  |  |  |  |  | |  | |  |  |  | 47.3 ± 20.3 months. | |  | First, it is retrospective subjected to selection bias. Second, the lack of a control group. measurement error. Fourth, no long‑term follow‑up was conducted to measure the persistence of effects. | | |
| 20 | Kim DH (24) | 2021 | South Korea | Retrospective cohort study | 3.85± 2.55 | 44.82% | 560 | Basic-type X(T) was classified when the measured deviation at distance was within 10 PD of the near deviation. | | | | | | | −18.5 ± 1.86 | | 29.15 ± 7.55 | 29.55 ± 7.95 |  |  |  |  | |  | |  |  |  | 9.55 ± 2.55 years | Student t-test and Pearson chi-square test. Kaplan–Meier survival analysis and the log rank test. Multivariate logistic regression. | | | | |
| 21 | Moon, Y (25) | 2023 | Republic of Korea | Retrospective review of the medical records | at surgery 7.5 ± 2.4 years | 43.90% | 196 |  |  |  |  | |  | |  | | 25.4 ± 6.0 | 30.1 ± 7.6 |  |  |  |  | |  | |  |  |  |  | paired t-test and analysis of variance | | Retrospective nature of the study. Second, manifest refraction was measured during the postoperative period, in contrast to preoperative cycloplegic refraction. This can result in a larger change in refractive error immediately after surgery than the actual change. Third, according to the type of surgery, the angle of deviation differed and that the amount of surgery was different, which may have influenced the result. Forth, since none of our subjects showed an oblique axis of astigmatism, we were not able to investigate the postoperative change in oblique axis of astigmatism. Finally, we have performed RP for IXT treatment since 2013. Therefore, there was a difference in the duration of surgery between the subgroups according to the type of surgery. | | |
| 22 | Alizadeh Y (26) | 2023 | Iran | Descriptive cross‑sectional | 2.25 ± 0.74 years | 38% | 106 |  |  |  |  | |  | | .. +1.34 ± 1.07 | |  |  |  |  |  |  | |  | |  |  |  | 4.39 ± 1.44 years | Shapiro‑Wilk test, T‑test. Analysis of variance (ANOVA), Linear regression. | | 1: moderate number of patients, 2: no measurement of stereopsis, 3: retrospective study, and 4: not categorizing the patients according to the over‑minus amount. | | |
| 23 | Han JY (27) | 2023 | Republic of Korea | Retrospective case control study | 6.57 ± 2.12 years | 50.51% | 388 |  |  |  |  | |  | | −0.74 ± 1.53 | | 20.88 ± 6.78 | 21.37 ± 6.52 |  |  |  |  | |  | | manifest refraction | |  | 58.7±12.85 weeks | Paired t-test, independent t-test, chisquare test, Fisher’s exact test, Mann–Whitney test, and repeated measures analysis of variance (ANOVA) | | Retrospective study. refractive errors in all patients were measured only using manifest refraction. the number of patients who did not undergo surgery was relatively smaller compared to those who received surgery. Fourth, other factors that may affect the progression of myopia—outdoor activities, degree of near work, and family history—were not reflected in the study. the entire follow-up period was relatively shorter than others. | | |
