# Supplementary material for: Exploring the Link: A Systematic Review and Meta‐Analysis on the Prevalence and Association Between Refractive Errors and Intermittent Exotropia
Source: Health Sci Rep. 2024 Dec 19;7(12):e70296. doi: 10.1002/hsr2.70296 (PMC11659191; doi:10.1002/hsr2.70296)
Supplement: Supplementary file 2 — Supporting information. [file HSR2-7-e70296-s002.docx]

| **Joanna Briggs Institute Checklist for Case Series – Criteria** |
| --- |
| 1. Were there clear criteria for inclusion in the case series? |
| 2. Was the condition measured in a standard, reliable way for all participants included in the case series? |
| 3. Were valid methods used for identification of the condition for all participants included in the case series? |
| 4. Did the case series have consecutive inclusion of participants? |
| 5. Did the case series have complete inclusion of participants? |
| 6. Was there clear reporting of the demographics of the participants in the study? |
| 7. Was there clear reporting of clinical information of the participants? |
| 8. Were the outcomes or follow up results of cases clearly reported? |
| 9. Was there clear reporting of the presenting site(s)/clinic(s) demographic information? |
| 10. Was statistical analysis appropriate? |
| **Responses Options**: Yes, No, Unclear, Not Applicable (NA) |
| **Quality Rating**: Poor 0 – 3; Fair 4 – 7; Good 8 – 10 |

| **Study** | **1** | **2** | **3** | **4** | **5** | **6** | **7** | **8** | **9** | **10** | **Rating** |
| --- | --- | --- | --- | --- | --- | --- | --- | --- | --- | --- | --- |
| Burton j. Kushner, MD – 1995 | Yes | Yes | Yes | Yes | No | No | No | Yes | Yes | Yes | 7 –Fair |
| Suh YW et al. – 2006 | Yes | Yes | Yes | Unclear | Yes | No | No | Yes | Yes | Unclear | 6 – Fair |
| Rowe FJ et al. – 2009 | Yes | Yes | Yes | Unclear | Yes | No | No | Yes | No | Yes | 6 – Fair |
| **NOHA S. EKDAWI** et al. – 2010 | Yes | Yes | Yes | Yes | Yes | Yes | Yes | Yes | Yes | Yes | 10 – Good |
| Jung JW  et al. – 2010 | Yes | Yes | Yes | Yes | Yes | No | Yes | No | No | Yes | 7 – Fair |
| Yang HK  et al. – 2011 | Yes | Yes | Yes | Yes | No | No | No | No | Yes | Yes | 6 – Fair |
| Lim SH et al. – 2012 | Yes | Yes | Yes | Unclear | Unclear | No | Yes | Yes | Yes | Yes | 7 – Fair |
| Jang JH et al. – 2012 | Yes | Yes | Yes | Unclear | Unclear | No | No | Yes | Yes | Yes | 6 – Fair |
| Hong SW et al. – 2012 | Yes | Yes | Yes | Unclear | Unclear | No | No | Yes | Yes | Yes | 6 – Fair |
| Wang, B et al. – 2014 | Yes | Yes | Yes | Unclear | Unclear | No | No | Yes | Yes | Yes | 6 – Fair |
| Kim MK  et al. – 2015 | Yes | Yes | Yes | Unclear | Unclear | No | No | Yes | Yes | Yes | 6 – Fair |
| Ha, S.-G et al. – 2016 | Yes | Yes | Yes | Unclear | Unclear | No | No | No | Yes | Yes | 5 – Fair |
| Yang, M. et al.2016 | Yes | Yes | Yes | Unclear | Unclear | No | Yes | Yes | Yes | Yes | 7 – Fair |
| Han D  et al. – 2018 | Yes | Yes | Yes | Unclear | Unclear | No | No | No | Yes | Yes | 5 – Fair |
| Ahn YJ et al. – 2019 | Yes | Yes | Yes | Unclear | Unclear | No | No | Yes | Yes | Yes | 6 – Fair |
| Lee D  et al. – 2019 | Yes | Yes | Yes | Unclear | Unclear | No | No | Yes | Yes | Yes | 6 – Fair |
| Lee DC et al. – 2020 | Yes | Yes | Yes | Unclear | No | No | No | Yes | Yes | Yes | 6 – Fair |
| Lee HJ et al. – 2020. | Yes | Yes | Yes | Unclear | No | No | No | Yes | Yes | Yes | 6 – Fair |
| Abri Aghdam et al. – 2021 | Yes | No | No | Unclear | Unclear | No | N0 | Yes | Yes | Yes | 4 – Fair |
| Kim DH et al. – 2021 | Yes | Yes | Yes | Unclear | Unclear | No | No | Yes | Yes | Yes | 6-Fair |
| Alizadeh Y et al. – 2023 | Yes | Yes | Yes | Unclear | Unclear | No | No | Yes | Yes | Yes | 6-Fair |
| Han JY et al. – 2023 | Yes | Yes | Yes | Unclear | Unclear | No | No | Yes | Yes | Yes | 6-Fair |
| Moon, Y et al. – 2023 | Yes | Yes | Yes | Unclear | Unclear | No | No | Yes | Yes | Yes | 6-Fair |
